# Occupational injury and job esteem in relation to burnout dimensions among nurses in Beijing: a cross-sectional study

**DOI:** 10.3389/fpsyg.2026.1940339

**Published:** 2026-09-18

**Authors:** Cunduo Jin, Haiyang Li, Wenyan Shi, Xianzong Ma, Wenyu Zhang

**Affiliations:** 1 Department of Nursing, The Seventh Medical Center of Chinese PLA General Hospital, Beijing, China; 2 Department of General Practice, The Seventh Medical Center of Chinese PLA General Hospital, Beijing, China; 3 Department of Cadre Diagnosis and Treatment, The Seventh Medical Center of Chinese PLA General Hospital, Beijing, China; 4 Senior Department of Gastroenterology, The First Medical Center of Chinese PLA General Hospital, Beijing, China; 5 School of Nursing, Capital Medical University, Beijing, China

**Keywords:** burnout, professional, job esteem, nurses, occupational health, occupational injuries

## Abstract

**Background:**

Burnout threatens nurses’ well-being and the sustainability of healthcare delivery. Occupational injury may contribute to burnout, whereas job esteem may provide an important professional resource; however, their associations with specific burnout dimensions remain unclear. This study examined the associations of occupational injury and job esteem with emotional exhaustion, depersonalization, and personal accomplishment among nurses.

**Methods:**

A cross-sectional survey was conducted from January 2023 to September 2024 among 3,654 nurses from 14 hospitals in Beijing. Burnout was assessed using the Maslach Burnout Inventory, job esteem using the Job Esteem Scale, and occupational injuries and exposures using a study-specific questionnaire. Three multivariable binary logistic regression models were fitted to examine factors independently associated with high emotional exhaustion, high depersonalization, and high personal accomplishment. Results are reported as adjusted odds ratios (aORs) with 95% confidence intervals (CIs).

**Results:**

Overall, 89.2% of participants had ever experienced an occupational injury or exposure, and 71.4% reported at least one event during the preceding 12 months. After adjustment, professional self-awareness (aOR = 0.580, 95% CI: 0.461–0.729; *p* < 0.001) and organizational respect and recognition (aOR = 0.659, 95% CI: 0.493–0.881; *p* = 0.005) were associated with lower odds of high emotional exhaustion, whereas social trust and respect was associated with higher odds (aOR = 1.332, 95% CI: 1.049–1.691; *p* = 0.019). All five job-esteem dimensions were associated with lower odds of high depersonalization (aORs = 0.488–0.793; all *p* ≤ 0.037). Organizational respect and recognition (aOR = 2.353, 95% CI: 1.499–3.695; *p* < 0.001), professional authority and future value (aOR = 2.892, 95% CI: 2.041–4.097; *p* < 0.001), and professional competence (aOR = 2.486, 95% CI: 1.398–4.420; *p* = 0.002) were associated with higher odds of high personal accomplishment. Psychological harm was associated with higher odds of high emotional exhaustion (aOR = 1.527, 95% CI: 1.261–1.847; *p* < 0.001) and high depersonalization (aOR = 1.410, 95% CI: 1.210–1.644; *p* < 0.001), and with lower odds of high personal accomplishment (aOR = 0.666, 95% CI: 0.492–0.900; *p* = 0.008). Increasing occupational injury or exposure frequency was primarily associated with higher odds of high emotional exhaustion.

**Conclusion:**

Occupational injuries and exposures and job esteem showed distinct associations with emotional exhaustion, depersonalization, and personal accomplishment among nurses. The findings highlight occupational safety, post-incident support, professional competence, and organizational recognition as relevant considerations in dimension-specific burnout management. Given the cross-sectional design, these associations should not be interpreted as causal.

## Introduction

The well-being of the nursing workforce is fundamental to healthcare quality, patient safety, and organizational effectiveness, as nurses provide continuous and direct patient care across healthcare settings (Zhang et al., 2021, 2022). However, job burnout has become a pervasive occupational health concern among nurses worldwide, with important consequences for both individual well-being and healthcare systems. Burnout is commonly conceptualized as a psychological response to chronic and insufficiently managed occupational stress and is characterized by three core dimensions: emotional exhaustion (EE), reflecting depleted emotional and physical energy; depersonalization (DP), characterized by detached or cynical attitudes toward one’s work and recipients of care; and reduced personal accomplishment (PA), reflecting diminished perceptions of professional competence and effectiveness (Zhang et al., 2021). Nurses may be particularly vulnerable to burnout because their work frequently involves high workloads, sustained emotional labor, unpredictable clinical demands, and irregular shift patterns (Aalto et al., 2018). Persistent exposure to these demands has been associated with burnout and a range of adverse physical and psychological outcomes among healthcare professionals (Aalto et al., 2018; Bakhamis et al., 2019). Beyond routine work demands, nurses practice in complex clinical environments that expose them to biological, chemical, physical, ergonomic, and psychosocial hazards (Fernández-Puerta et al., 2025; Khairallah et al., 2025). Although occupational injury has traditionally referred primarily to direct physical harm, such as needlestick injuries, pathogen exposure, and musculoskeletal injury, contemporary occupational-health frameworks increasingly recognize the broader and potentially cumulative consequences of workplace hazards (Malik et al., 2023). Evidence from healthcare settings indicates a substantial burden of occupational injuries and hazardous exposures, particularly during public health emergencies, when intensified workloads, infection risks, and psychological pressures may coexist (Al-Ghunaim et al., 2021). Repeated or cumulative exposure to occupational hazards has also been associated with adverse health outcomes, including burnout (Campione and Henschke, 2025; Khairallah et al., 2025). Nevertheless, the associations of different types and frequencies of occupational injury with the individual dimensions of nurse burnout remain insufficiently understood.

Alongside occupational demands, psychological and professional resources may play an important role in nurses’ responses to workplace stress. Job esteem, defined as the respect, value, and sense of pride individuals derive from their professional role, is particularly relevant to nursing because the profession is closely connected with ethical caregiving, professional competence, and social responsibility (Yu et al., 2023). Job esteem contributes to nurses’ professional self-concept, attitudes, and work-related behaviors (Montoya and Pinter, 2024). Higher job esteem has been associated with more favorable professional attitudes and functioning, whereas insufficient professional recognition and diminished perceptions of occupational value may contribute to emotional detachment and intentions to leave nursing (Ghaleh et al., 2024). Importantly, job esteem is a multidimensional construct encompassing professional self-awareness, social trust and respect, organizational respect and recognition, professional authority and future value, and professional competence (Choi and Jung, 2020). These dimensions reflect distinct personal, organizational, and social resources and may therefore have different relationships with EE, DP, and PA. However, these dimension-specific associations have received limited empirical attention.

The Conservation of Resources (COR) theory provides the primary framework for understanding the relationships among occupational injury, job esteem, and burnout (Hobfoll, 1989). COR theory proposes that individuals seek to acquire, retain, and protect valued resources and experience stress when those resources are threatened, lost, or inadequately replenished (Hobfoll, 1989). From this perspective, occupational injury and repeated exposure to workplace hazards may deplete nurses’ physical energy, psychological security, time, and coping capacity. Job esteem, by contrast, may function as a professional resource that reinforces perceived competence, organizational recognition, professional meaning, and social value. Occupational injury would therefore be expected to correspond to less favorable burnout outcomes, whereas stronger job esteem would be expected to correspond to lower EE and DP and higher PA. At the organizational level, the Resource-Based View (RBV) offers a complementary perspective by conceptualizing human capital as a valuable resource that supports sustained organizational functioning (Barney, 1991; Clark et al., 2013). Nurses’ clinical expertise, accumulated experience, professional commitment, and relational knowledge are important organizational resources that support the continuity and quality of patient care. Occupational injury and burnout may erode these resources, whereas occupational protection, professional recognition, and development opportunities may help preserve them. Accordingly, the RBV perspective helps situate the relationships examined in this study within the broader context of nursing workforce sustainability and organizational capacity.

Guided primarily by COR theory and informed by the RBV perspective, this study aimed to assess the levels of job burnout, occupational injury, and job esteem among Chinese nurses and to examine the independent associations of occupational injury and specific dimensions of job esteem with EE, DP, and PA. By considering resource-depleting occupational experiences and potentially protective professional resources within a common framework, the study may provide a more integrated understanding of nurse burnout and inform targeted strategies for occupational safety, professional support, and nursing workforce management.

## Methods

### Study design and setting

This cross-sectional study was conducted among nurses in Beijing from January 2023 to September 2024. The study protocol, including the questionnaire structure and methodology, underwent three rounds of rigorous expert review by specialists from the Chinese PLA General Hospital, Peking Union Medical College, and Capital Medical University to ensure content validity. Eligible participants were registered nurses employed full-time in clinical settings within Beijing. Inclusion criteria were defined as follows: (1) possession of a valid Chinese nursing license; (2) a minimum of 2 years of full-time clinical nursing experience; and (3) prior involvement in the management of acute public health challenges. Exclusion criteria comprised: (1) refusal to participate; (2) absence of valid licensure; (3) a diagnosed psychiatric disorder or severe chronic condition potentially impairing work capacity (e.g., severe depression or uncontrolled cardiovascular disease); and (4) holding non-clinical positions (e.g., exclusively administrative roles). This study was conducted in accordance with the Declaration of Helsinki and was approved by the Medical Ethics Committee of Capital Medical University (Approval No. Z2023SY051). Electronic informed consent was obtained from all participants before they accessed the questionnaire.

### Data collection

A multistage purposive cluster sampling approach was used. Hospital grade (tertiary, secondary, or primary), geographic location (urban, suburban, or exurban), and institutional ownership (public or private) were considered when selecting hospitals to include institutions with different characteristics. Fourteen hospitals were purposively selected based on these predefined institutional characteristics and the feasibility of study implementation. These hospital categories were used to guide institutional selection rather than for proportional sample allocation. Therefore, no separate sample size was predetermined for each category.

The overall minimum sample size was calculated using the formula: *N* = (Uασ/δ)^2^. where N represents the minimum required sample size, Uα is the standard normal deviate corresponding to the specified significance level, σ is the estimated standard deviation, and δ is the allowable error. Based on a standard deviation (σ) of 1.15, a two-sided significance level of 0.05 (Uα = 1.96), and an allowable error (δ) of 0.066, the minimum required sample size was 1,166. After accounting for a potential 10% rate of nonresponse or invalid questionnaires, the recruitment target was approximately 1,296. Because the number of eligible nurses substantially exceeded the minimum requirement, all eligible nurses in the participating hospitals were invited to participate to increase the precision of the estimates.

Data collection was conducted using the SO JUMP platform.^1^ An anonymous survey link, configured to allow only one submission per respondent, was distributed to all eligible nurses via institutional communication channels and professional networks. Prior to participation, all individuals received information about the study’s purpose and procedures and provided electronic informed consent, which they were required to confirm before starting the survey. Participation was entirely voluntary, and respondents could withdraw at any time without providing a reason.

A total of 3,900 eligible nurses from the 14 participating hospitals were approached and invited to participate. Of these, 3,675 provided electronic informed consent and submitted the questionnaire, corresponding to a participation rate of 96.4%. The submitted questionnaires were subsequently subjected to the prespecified data-quality screening procedures described below.

### Questionnaire development

The survey comprised four parts. Part one aimed to gather demographic information, including gender, age, marital status, average monthly income, Satisfaction with personal financial situation, education level, positional titles, hospital level, hospital type and labor relationship.

Part two was designed to assess occupational exposures and protection, including the following items: Have you ever experienced an occupational injury (yes and no); Type of occupational Injury (disinfectants, psychological harm, infection and physical injury); How frequently have you been exposed to occupational injuries? (times in recent 1 year) (0, 1–5, 6–10, 11–15, ≥16); Perceived risk of occupational exposure (none, low, moderate, high, very high); Do you need occupational safety and protection training? (yes and no); Perceived hospital emphasis on occupational safety (none, low, moderate, high, very high).

Part three was provided to investigate the job burnout among nurses using the Maslach Burnout Inventory (MBI) (Alacacioglu et al., 2009), which contain 22 items and had three subscales: EE (9 items), DP (5 items) and PA (8 items). Each item was rated on a 5-point frequency scale (never, a few times a year, a few times a month, a few times a week, and every day). Higher EE and DP scores indicated more severe symptoms, whereas a higher PA score indicated a stronger sense of professional accomplishment (Pan et al., 2026).

Part four assessed job esteem using the Chinese version of the Job Esteem Scale (JES). The original Korean-language instrument was developed by Choi and Jung (2020), and the Chinese version was translated and culturally adapted by Shi and Shen (2021). The scale comprises 25 items across five dimensions: professional self-awareness (4 items), social trust and respect (3 items), organizational respect and recognition (4 items), professional authority and future value (4 items), and professional competence (10 items). Items are rated on a 5-point Likert scale ranging from 1 (completely disagree) to 5 (completely agree), with higher scores indicating greater job esteem. In the present study, Cronbach’s alpha was 0.921 for the total scale. The Cronbach’s alpha coefficients for the five dimensions were 0.890 for professional self-awareness, 0.888 for social trust and respect, 0.877 for organizational respect and recognition, 0.940 for professional authority and future value, and 0.960 for professional competence.

### Data management and quality control

Given the use of an online survey platform, procedural controls were implemented to ensure data integrity. The survey was configured with mandatory fields to prevent missing data at the source. Following data collection, raw data were exported directly from the SO JUMP platform into Microsoft Excel, eliminating manual entry errors. A rigorous data cleaning protocol was applied to identify invalid submissions based on two criteria: a completion time of <120 s, suggesting insufficient attention to item content; and discernible response patterns. These invalid responses were excluded from the final dataset prior to statistical analysis. All 3,675 submitted questionnaires were checked before analysis. Twenty-one were excluded: 9 had a completion time of <120 s, 7 contained logical inconsistencies between related items, and 5 showed repetitive or invariant response patterns. Each excluded questionnaire was assigned to one exclusion category. The final analysis included 3,654 valid questionnaires, giving a valid-questionnaire rate of 99.4%.

Rigorous quality control measures were implemented throughout the data management process. To mitigate common method bias (CMB), procedural remedies included ensuring respondent anonymity, randomizing item order, and separating predictor and outcome variables within the survey design. Statistical assessment using Harman’s single-factor test revealed that the first factor accounted for 26.2% of the total variance, well below the 40% threshold, indicating that CMB did not substantially affect the results. Following data collection, a systematic cleaning protocol was applied. Invalid questionnaires—characterized by logical inconsistencies or completion times significantly below the pilot average—were excluded. A random sample of 20% of the dataset underwent logical verification against source parameters to ensure accuracy. Data processing was conducted using standard validation algorithms to identify and correct any potential outliers or errors.

### Statistical analysis

All statistical analyses were performed using SPSS version 23.0 (IBM Corp., Armonk, NY, USA). Categorical variables were summarized as frequencies and percentages. Continuous variables meeting the assumptions of parametric analysis were summarized as means and standard deviations (SDs), and between-group differences were assessed using independent-samples *t*-tests. Internal consistency was evaluated using Cronbach’s alpha. Pearson’s correlation coefficients were calculated to assess the linear associations between the five continuous JES dimension scores and the continuous EE, DP, and PA scores. All correlation tests were two-sided.

Three separate multivariable binary logistic regression models were fitted to examine factors associated with EE, DP, and PA. The cutoff points were selected with reference to Pan et al. (2026). EE scores ≥27, DP scores ≥10, and PA scores ≥33 were coded as 1, with scores below the corresponding cutoff coded as 0. The same explanatory variables were entered into each model: the five JES dimension scores; previous experience of occupational injury or exposure; four indicators of exposure type (disinfectant-related exposure, psychological harm, exposure to infectious agents, and physical injury); frequency of occupational injury or exposure; perceived risk of occupational exposure; need for occupational safety and protection training; and perceived hospital emphasis on occupational safety. These variables were selected based on their substantive relevance to the study objectives rather than their statistical significance in univariable analyses. The five JES dimension scores were entered as continuous variables. Previous experience of occupational injury or exposure and need for occupational safety and protection training were entered as binary variables. Because respondents could report multiple exposure types, the four exposure types were entered as separate binary indicators, with absence of the corresponding exposure type as the reference category. Exposure frequency, perceived exposure risk, and perceived hospital emphasis on occupational safety were entered as categorical variables using indicator coding; the reference categories are reported in the regression table. Results are presented as adjusted odds ratios (aORs) with 95% confidence intervals (CIs), with each aOR adjusted for all other explanatory variables included in the same model. All tests were two-sided, and *p* value < 0.05 was considered statistically significant.

## Results

### Demographic and professional characteristics of the participants

A total of 3,654 nurses were included in the study. Their demographic and professional characteristics are presented in Table 1. Most participants were female (95.3%) and aged 45 years or younger (93.8%). Overall, 78.1% had a bachelor’s degree or higher. The most common professional title categories were nurse practitioner (42.4%) and nurse-in-charge (30.7%). Most participants worked in tertiary hospitals (82.8%) and general hospitals (90.6%). Contract-based employment was the most common employment arrangement (64.0%). In addition, 72.5% reported an average monthly income exceeding RMB 8,000.

**Table 1:** Demographic and professional characteristics of the participants (*N* = 3,654).

Characteristics	*n* (%)
Gender	
Female	3,482 (95.3)
Male	172 (4.7)
Age (years)	
≤45	3,427 (93.8)
≥46	227 (6.2)
Marital status	
Unmarried	1,383 (37.8)
Married	2,215 (60.6)
Divorced	46 (1.3)
Other	10 (0.3)
Average monthly income (Renminbi, RMB, Yuan)	
≤8,000	1,005 (27.5)
≥8,001	2,649 (72.5)
Satisfaction with personal financial situation	
Very satisfied	377 (10.3)
Satisfied	943 (25.8)
Neutral	1,706 (46.7)
Dissatisfied	470 (12.9)
Very dissatisfied	158 (4.3)
Educational level	
Secondary vocational education	39 (1.1)
Higher vocational education	763 (20.9)
Bachelor’s degree	2,804 (76.7)
Master’s degree	48 (1.3)
Professional title	
Nurse	880 (24.1)
Nurse practitioner	1,550 (42.4)
Nurse-in-charge	1,121 (30.7)
Associate chief nurse	92 (2.5)
Chief nurse	11 (0.3)
Hospital grade	
Primary hospital	92 (2.5)
Secondary hospital	289 (7.9)
Tertiary hospital	3,026 (82.8)
Other	247 (6.8)
Hospital category	
General	3,312 (90.6)
Non-general	342 (9.4)
Labor relationship	
Permanent nurse	994 (27.2)
Contract nurse	2,339 (64.0)
Other	321 (8.8)

### Occupational exposure and protection

The participants’ occupational exposure and protection characteristics are presented in Table 2. Overall, 3,259 nurses (89.2%) reported having experienced an occupational injury or exposure at some point. During the preceding 12 months, 2,608 participants (71.4%) experienced at least one occupational injury or exposure event. Specifically, 2,325 (63.6%) reported 1–5 events, whereas 1,046 (28.6%) reported no such events. Among the 3,259 nurses who reported ever experiencing an occupational injury or exposure, exposure to infectious agents was the most frequently reported type (2,641, 81.0%), followed by psychological harm (1,584, 48.6%), disinfectant-related exposure (1,483, 45.5%), and physical injury (1,120, 34.4%). Multiple responses were permitted.

**Table 2:** Occupational exposure and protection characteristics among nurses (N = 3,654).

Variables	Frequency, *n* (%)
Have you ever experienced an occupational injury or exposure?	
Yes	3,259 (89.2)
No	395 (10.8)
Type of occupational injury or exposure^#^	
Disinfectant-related exposure	1,483 (45.5)
Psychological harm	1,584 (48.6)
Exposure to infectious agents	2,641 (81.0)
Physical injury	1,120 (34.4)
How frequently have you been exposed to occupational injuries (times in recent 1 year)?	
0	1,046 (28.6)
1–5	2,325 (63.6)
6–10	139 (3.8)
11–15	30 (0.8)
≥16	114 (3.1)
Perceived risk of occupational exposure	
None	464 (12.7)
Low	1,062 (29.1)
Moderate	1,253 (34.3)
High	682 (18.7)
Very high	193 (5.3)
Do you need occupational safety and protection training?	
Yes	3,203 (87.7)
No	451 (12.3)
Perceived hospital emphasis on occupational safety	
None	72 (2.0)
Low	322 (8.8)
Moderate	955 (26.1)
High	1,445 (39.5)
Very high	860 (23.5)

# Among the 3,259 nurses who reported ever experiencing an occupational injury or exposure, multiple responses were permitted; therefore, percentages do not sum to 100%.

Regarding perceived occupational exposure risk, the largest proportion of participants rated the risk as moderate (1,253, 34.3%). In addition, 3,203 participants (87.7%) indicated a need for occupational safety and protection training. Overall, 2,305 participants (63.1%) perceived their hospital’s emphasis on occupational safety as high (1,445, 39.5%) or very high (860, 23.5%).

### Descriptive statistics of job esteem and job burnout

Descriptive statistics for job esteem and job burnout are presented in Table 3. Among the five dimensions of job esteem, professional self-awareness had the highest mean item score (3.36 ± 0.50), followed by social trust and respect (3.35 ± 0.54), professional competence (3.26 ± 0.48), and organizational respect and recognition (3.12 ± 0.60). Professional authority and future value had the lowest mean item score (2.83 ± 0.69). Regarding job burnout, the mean item scores for EE, DP, and PA were 2.37 ± 0.65, 2.01 ± 0.64, and 2.89 ± 0.53, respectively. Their corresponding mean total scores were 21.33 ± 5.88, 10.06 ± 3.20, and 23.12 ± 4.24, respectively. Regarding the categorical distributions, 2,957 participants (80.9%) had EE scores <27 and 697 (19.1%) had scores ≥27; 1,506 (41.2%) had DP scores <10 and 2,148 (58.8%) had scores ≥10; and 258 (7.1%) had PA scores <33 and 3,396 (92.9%) had scores ≥33. Percentages were calculated using the full analytical sample (N = 3,654) as the denominator.

**Table 3:** Descriptive statistics for job esteem and job burnout (*N* = 3,654).

Dimension	Total score, mean ± SD	Mean item score, mean ± SD
Job esteem		
Professional self-awareness	13.44 ± 2.00	3.36 ± 0.50
Social trust and respect	10.04 ± 1.63	3.35 ± 0.54
Organizational respect and recognition	12.49 ± 2.40	3.12 ± 0.60
Professional authority and future value	11.32 ± 2.74	2.83 ± 0.69
Professional competence	32.59 ± 4.77	3.26 ± 0.48
Job burnout		
Emotional exhaustion	21.33 ± 5.88	2.37 ± 0.65
Depersonalization	10.06 ± 3.20	2.01 ± 0.64
Personal accomplishment	23.12 ± 4.24	2.89 ± 0.53

Higher scores for emotional exhaustion and depersonalization and lower scores for personal accomplishment indicate higher levels of job burnout.

### Correlations between job esteem and job burnout dimensions

Pearson correlation analysis showed that all five dimensions of job esteem were significantly negatively correlated with EE and DP (*r* = −0.456 to −0.283, all *p* < 0.01) and significantly positively correlated with PA (*r* = 0.296 to 0.373, all *p* < 0.01). Professional authority and future value showed the strongest negative correlation with EE (*r* = −0.456), whereas professional competence showed the strongest positive correlation with PA (*r* = 0.373; Table 4).

**Table 4:** Pearson correlations between job esteem dimensions and job burnout dimensions (*N* = 3,654).

Dimension	Professional self-awareness	Social trust and respect	Organizational respect and recognition	Professional authority and future value	Professional competence
Emotional exhaustion	−0.352	−0.283	−0.398	−0.456	−0.354
Depersonalization	−0.351	−0.291	−0.304	−0.288	−0.331
Personal accomplishment	0.361	0.296	0.314	0.315	0.373

All correlations were statistically significant at *p* < 0.01 (two-tailed). Pearson’s *r* values are presented.

### Factors associated with job burnout dimensions

Multivariable binary logistic regression analyses were conducted to identify factors associated with EE, DP, and PA (Table 5). Higher professional self-awareness (aOR = 0.580, 95% CI: 0.461–0.729, *p* < 0.001) and organizational respect and recognition (aOR = 0.659, 95% CI: 0.493–0.881, *p* = 0.005) were associated with lower odds of high EE. In contrast, social trust and respect was associated with higher odds of high EE (aOR = 1.332, 95% CI: 1.049–1.691, *p* = 0.019).

**Table 5:** Factors associated with the dimensions of job burnout in multivariable binary logistic regression models (*N* = 3,654).

Variables	Emotional exhaustion		Depersonalization		Personal accomplishment	
	aOR (95% CI)	*p* value	aOR (95% CI)	*p* value	aOR (95% CI)	*p* value
Job esteem^a^						
Professional self-awareness	0.580 (0.461–0.729)	<0.001	0.488 (0.404–0.589)	<0.001	1.396 (0.832–2.343)	0.206
Social trust and respect	1.332 (1.049–1.691)	0.019	0.696 (0.570–0.849)	<0.001	1.280 (0.763–2.147)	0.349
Organizational respect and recognition	0.659 (0.493–0.881)	0.005	0.793 (0.637–0.986)	0.037	2.353 (1.499–3.695)	<0.001
Professional authority and future value	0.892 (0.657–1.211)	0.463	0.773 (0.622–0.961)	0.020	2.892 (2.041–4.097)	<0.001
Professional competence	0.795 (0.614–1.030)	0.083	0.590 (0.480–0.727)	<0.001	2.486 (1.398–4.420)	0.002
Have you ever experienced an occupational injury or exposure?						
Yes	Reference		Reference		Reference	
No	0.827 (0.603–1.134)	0.238	0.969 (0.765–1.230)	0.798	1.247 (0.829–1.877)	0.290
Type of occupational injury or exposure^b^						
Disinfectant-related exposure	0.981 (0.809–1.189)	0.841	0.973 (0.833–1.136)	0.730	1.079 (0.801–1.452)	0.617
Psychological harm	1.527 (1.261–1.847)	<0.001	1.410 (1.210–1.644)	<0.001	0.666 (0.492–0.900)	0.008
Exposure to infectious agents	1.024 (0.815–1.286)	0.842	1.113 (0.925–1.339)	0.257	0.848 (0.597–1.204)	0.357
Physical injury	1.376 (1.133–1.670)	0.001	1.154 (0.979–1.361)	0.088	0.799 (0.577–1.105)	0.175
How frequently have you been exposed to occupational injuries?						
0	Reference		Reference		Reference	
1–5	1.288 (1.025–1.618)	0.030	1.179 (0.994–1.397)	0.058	0.866 (0.637–1.177)	0.357
6–10	2.573 (1.702–3.890)	< 0.001	1.226 (0.825–1.822)	0.313	0.803 (0.335–1.923)	0.623
11–15	2.755 (1.253–6.059)	0.012	2.770 (1.098–6.988)	0.031	0.493 (0.065–3.765)	0.495
≥16	2.097 (1.323–3.322)	0.002	1.148 (0.753–1.751)	0.520	1.249 (0.570–2.735)	0.579
Perceived risk of occupational exposure						
None	Reference		Reference		Reference	
Low	0.735 (0.540–1.001)	0.051	1.204 (0.968–1.498)	0.095	0.712 (0.498–1.016)	0.061
Moderate	1.147 (0.861–1.529)	0.349	1.816 (1.464–2.252)	<0.001	0.415 (0.285–0.606)	<0.001
High	2.065 (1.531–2.786)	<0.001	2.133 (1.675–2.717)	<0.001	0.436 (0.282–0.674)	<0.001
Very high	3.498 (2.391–5.118)	<0.001	1.761 (1.250–2.482)	0.001	0.459 (0.234–0.898)	0.023
Do you need occupational safety and protection training?						
Yes	Reference		Reference		Reference	
No	1.229 (0.962–1.570)	0.100	1.028 (0.834–1.266)	0.798	0.803 (0.521–1.236)	0.318
Perceived hospital emphasis on occupational safety						
None	Reference		Reference		Reference	
Low	1.932 (1.018–3.669)	0.044	2.379 (1.388–4.077)	0.002	0.581 (0.193–1.749)	0.335
Moderate	1.100 (0.593–2.039)	0.762	1.562 (0.955–2.554)	0.075	0.526 (0.195–1.414)	0.203
High	0.681 (0.369–1.259)	0.221	1.114 (0.687–1.806)	0.662	0.995 (0.386–2.562)	0.991
Very high	0.692 (0.370–1.294)	0.249	0.568 (0.348–0.927)	0.024	2.403 (0.939–6.147)	0.067

Note. aOR = adjusted odds ratio; CI = confidence interval. 𝐿or each job esteem dimension, the aOR represents the change in odds associated with a one-point increase in the corresponding mean item score. 𝑿or each type of occupational injury or exposure, the reference category was no exposure. Emotional exhaustion scores ≥27 were coded as 1 and scores <27 as 0; depersonalization scores ≥10 were coded as 1 and scores <10 as 0; and personal accomplishment scores ≥33 were coded as 1 and scores <33 as 0. For each job esteem dimension, the aOR represents the change in odds associated with a one-point increase in the corresponding mean item score.

All five job esteem dimensions were associated with lower odds of high DP: professional self-awareness (aOR = 0.488, 95% CI: 0.404–0.589, *p* < 0.001), social trust and respect (aOR = 0.696, 95% CI: 0.570–0.849, *p* < 0.001), organizational respect and recognition (aOR = 0.793, 95% CI: 0.637–0.986, *p* = 0.037), professional authority and future value (aOR = 0.773, 95% CI: 0.622–0.961, *p* = 0.020), and professional competence (aOR = 0.590, 95% CI: 0.480–0.727, *p* < 0.001). Organizational respect and recognition (aOR = 2.353, 95% CI: 1.499–3.695, *p* < 0.001), professional authority and future value (aOR = 2.892, 95% CI: 2.041–4.097, *p* < 0.001), and professional competence (aOR = 2.486, 95% CI: 1.398–4.420, *p* = 0.002) were associated with higher odds of having a PA score ≥33.

Psychological harm was associated with higher odds of high EE (aOR = 1.527, 95% CI: 1.261–1.847, *p* < 0.001) and high DP (aOR = 1.410, 95% CI: 1.210–1.644, *p* < 0.001), as well as lower odds of having a PA score ≥33 (aOR = 0.666, 95% CI: 0.492–0.900, *p* = 0.008). Physical injury was associated with higher odds of high EE (aOR = 1.376, 95% CI: 1.133–1.670, *p* = 0.001). Disinfectant-related exposure and exposure to infectious agents were not significantly associated with any of the three outcomes.

Compared with no occupational injury or exposure events in the preceding 12 months, each of the following exposure-frequency categories was associated with higher odds of high EE: 1–5 events (aOR = 1.288, 95% CI: 1.025–1.618, *p* = 0.030), 6–10 events (aOR = 2.573, 95% CI: 1.702–3.890, *p* < 0.001), 11–15 events (aOR = 2.755, 95% CI: 1.253–6.059, *p* = 0.012), and ≥16 events (aOR = 2.097, 95% CI: 1.323–3.322, *p* = 0.002) was associated with higher odds of high EE. Only the 11–15-event category was associated with higher odds of high DP (aOR = 2.770, 95% CI: 1.098–6.988, *p* = 0.031). Exposure frequency was not significantly associated with PA. Compared with nurses reporting no perceived occupational exposure risk, those reporting high and very high perceived risk had higher odds of high EE. Moderate, high, and very high perceived risk were associated with higher odds of high DP and lower odds of high PA. Low perceived risk was not significantly associated with any of the three outcomes.

Regarding perceived hospital emphasis on occupational safety, low emphasis was associated with higher odds of high EE (aOR = 1.932, 95% CI: 1.018–3.669, *p* = 0.044) and high DP (aOR = 2.379, 95% CI: 1.388–4.077, *p* = 0.002), whereas very high emphasis was associated with lower odds of high DP (aOR = 0.568, 95% CI: 0.348–0.927, *p* = 0.024). Neither having ever experienced an occupational injury or exposure nor the perceived need for occupational safety and protection training was significantly associated with any of the three outcomes.

## Discussion

This large-sample study examined the relationships of occupational injuries and job esteem with the three dimensions of burnout among Chinese nurses. Occupational injuries and specific dimensions of job esteem were associated differently with EE, DP, and PA, indicating that these dimensions should be considered separately. From the RBV (Barney, 1991; Clark et al., 2013), nurses are an important source of human capital because their clinical knowledge, technical expertise, and interpersonal skills contribute directly to care quality and hospital performance. Burnout may reduce the retention and effective use of these capabilities, while a safe work environment and positive job esteem may help preserve them.

The participants were relatively young and well educated, reflecting changes in the composition of the Chinese nursing workforce. The proportion of nurses with a bachelor’s degree was higher than that reported in earlier studies (Zhang et al., 2021, 2022), possibly reflecting recent reforms in nursing education. The distribution of professional titles was broadly consistent with the hierarchical structure of the Chinese healthcare workforce (Mao et al., 2025). However, the small proportion of nurses with senior professional titles may reflect challenges in career progression, consistent with recent evidence showing multiple barriers to promotion to senior professional ranks among Chinese clinical nurses (Zheng et al., 2026). The relatively high-income level and concentration of participants in tertiary hospital and general hospitals are likely related to the Beijing-based sample, given the city’s economic development and concentration of healthcare resources.

The levels of EE and DP in our sample warrant attention. Previous studies of Chinese nurses have also reported considerable levels of EE and DP, although estimates vary across study populations and settings (Wang et al., 2015; Li et al., 2024a; Li et al., 2024b). For example, a recent national study of 4,865 nurses found that 50.7% had moderate or high EE and 32.8% had moderate or high DP (Li et al., 2024a; Li et al., 2024b). The persistence of DP is particularly relevant to clinical practice, as nurse burnout has been associated with lower patient safety, lower patient satisfaction, and poorer quality of care (Li et al., 2024a; Li et al., 2024b). Low PA was also common in our sample. This finding is consistent with recent evidence from China, where 80.4% of nurses were classified as having low or moderate PA (Li et al., 2024a; Li et al., 2024b). Taken together, these findings suggest that burnout in nurses involves not only emotional strain but also reduced professional accomplishment, both of which should be considered when developing workforce support strategies.

All dimensions of job esteem were negatively correlated with EE and DP and positively correlated with PA. These findings are compatible with COR theory, which considers valued personal and work-related resources relevant to how individuals respond to occupational stress (Hobfoll, 1989). Professional authority and future value had the strongest negative correlation with EE, whereas Professional Competence had the strongest positive correlation with PA. Thus, different aspects of job esteem may relate to different components of burnout. Perceived long-term professional value may reduce exhaustion associated with a lack of meaning, while confidence in professional competence may contribute more directly to a sense of accomplishment.

The multivariable analyses revealed a clear difference across burnout dimensions. EE and DP were predominantly adverse occupational exposures, whereas PA was more strongly related to professional and organizational resources, particularly professional competence and organizational recognition. Viewed through the lens of COR theory (Hobfoll, 1989), these findings suggest that burnout may not represent a uniform response to the work environment. Rather, exhaustion and DP appear to be more closely aligned with resource depletion, while professional accomplishment is more closely linked to resources that reinforce competence, recognition, and professional value. Previous research among Chinese nurses has similarly identified workplace violence and perceived organizational support as important components of the occupational context associated with burnout (Liu et al., 2018). The present findings extend this perspective by showing that occupational risks and job-esteem resources were associated with different dimensions of burnout. This distinction is relevant to workforce management, as reducing adverse occupational exposures and strengthening professional resources represent complementary, rather than interchangeable, aspects of addressing nurse burnout.

A notable finding was the change in the association between Social Trust and Respect and EE after multivariable adjustment. Although this dimension was negatively correlated with EE in the bivariate analysis, higher social trust and respect scores were associated with greater odds of EE after adjustment for the other factors. This finding contrasts with previous evidence indicating that recognition and respect at work are generally associated with lower burnout, particularly lower EE (Renger et al., 2020). One interpretation is that the meaning of social recognition may change when it is considered alongside other dimensions of job esteem. Recognition can affirm the social value of nursing, but it may also carry expectations regarding commitment, emotional responsiveness, and standards of care. In settings where nurses face high workloads or limited organizational resources, these expectations may become difficult to reconcile with what can realistically be delivered. Emotional labor may be relevant in this context, as greater emotional labor has been associated with higher burnout among nurses (Kim et al., 2022). Thus, professional respect may be most beneficial when recognition of nurses is matched by working conditions that enable them to meet the professional expectations attached to that recognition. Improving the social standing of nursing should therefore be accompanied by adequate staffing, manageable workloads, realistic role expectations, and organizational support.

Psychological harm was associated with all three dimensions of burnout, while more frequent occupational injuries were consistently associated with less favorable burnout outcomes. Taken together, these findings highlight a broader dimension of occupational safety that extends beyond the immediate physical consequences of individual incidents. This pattern is consistent with meta-analytic evidence linking workplace violence among nurses with both burnout and post-traumatic stress symptoms (Wang et al., 2022). Importantly, the association with injury frequency suggests that the occupational burden may accumulate across repeated adverse events rather than be adequately captured by the severity of any single incident. Psychological consequences and recurrent exposure should therefore be considered alongside physical injury in occupational safety management. This requires not only measures to prevent workplace violence, but also accessible reporting procedures, systematic review of recurrent incidents, and structured post-incident support (Yew et al., 2026). Such an approach may help identify patterns of repeated exposure that could otherwise remain obscured when incidents are considered in isolation.

Associations between job-esteem dimensions and burnout suggest that professional resources may have different functions. The inverse association between professional self-awareness and DP may reflect the role of a coherent professional identity in reducing detachment and cynicism (Sabancıogullari and Dogan, 2015). PA was more strongly associated with professional competence, prestige, and organizational recognition than with managerial prioritization of safety, suggesting that professional accomplishment may depend particularly on opportunities to develop and use expertise and to receive recognition. Although workplace safety remains essential, it may address a different aspect of nurses’ working conditions (Mudallal et al., 2017). Individual stress-management programs should therefore complement, rather than replace, organizational efforts to reduce occupational risks and support professional development and recognition.

The observed associations also have implications for hospital workforce strategy. From an RBV perspective (Barney, 1991; Clark et al., 2013), retaining and developing nursing expertise is relevant to the management of strategically valuable organizational resources. Burnout is associated with greater turnover intention among nurses and with poorer patient safety, satisfaction, and quality outcomes (Özkan, 2022; Li et al., 2024a; Li et al., 2024b). Evidence on nurse retention likewise supports the importance of supportive work environments, leadership, and career-development strategies (Kiptulon et al., 2025). These findings support incorporating occupational safety and professional support into workforce planning and service-quality management, rather than treating them solely as employee welfare concerns.

### Limitation

Several limitations should be acknowledged. First, this cross-sectional study was conducted in Beijing, a resource-rich metropolitan area, and participating hospitals were purposively selected; therefore, the findings may not be generalizable to nurses in rural or resource-constrained settings. Second, the use of self-reported measures may have introduced recall, social desirability, and common-method biases, despite the procedural measures taken to minimize these effects. Third, the cross-sectional design precludes conclusions regarding temporality or causality among occupational injuries, job esteem, and burnout. Future multicenter longitudinal and intervention studies involving more diverse healthcare settings are needed to examine the temporal and potentially causal relationships among these factors.

### Managerial and practical implications

The findings suggest that burnout management should distinguish among EE, DP, and PA. Healthcare organizations should prioritize the prevention of psychological harm and repeated occupational injuries through risk control, non-punitive reporting, and timely post-incident support. In parallel, managers should strengthen nurses’ professional resources by promoting competency development, career advancement, professional identity, and organizational recognition. Efforts to enhance the social status of nursing should also be accompanied by adequate staffing, manageable workloads, and realistic role expectations to avoid creating additional emotional pressure. Thus, organizational strategies should integrate occupational safety with professional development rather than rely solely on individual-level wellness programs. Given the cross-sectional design, these implications should be interpreted cautiously and evaluated in future longitudinal or intervention studies.

## Conclusion

In conclusion, occupational injuries and job esteem showed distinct associations with EE, DP, and PA among Beijing nurses. Psychological harm was associated with less favorable outcomes across all three burnout dimensions, while repeated occupational injury or exposure events were primarily associated with EE. Job-esteem dimensions were differentially associated with the three outcomes, with professional self-awareness and organizational recognition associated with lower odds of EE, all five dimensions associated with lower odds of DP, and organizational recognition, professional authority and future value, and professional competence associated with higher PA. These findings support dimension-specific assessment of burnout and highlight occupational safety and professional resources as relevant considerations for nursing workforce management. Given the cross-sectional design, these associations should not be interpreted as causal and should be further examined in longitudinal and intervention studies.

## Data Availability

The original contributions presented in the study are included in the article/supplementary material, further inquiries can be directed to the corresponding author.

## Footnotes

1
https://www.wjx.cn/
